# Endotoxin-induced Ferroptosis-associated Leukocyte Injury and DAMP Release in Sepsis: An *in Vitro* Study

**DOI:** 10.14789/ejmj.JMJ26-0044-OA

**Published:** 2026-06-12

**Authors:** JERROLD H. LEVY, NAO UMEI, YUTAKA KONDO, ISAO NAGAOKA, TOSHIAKI IBA

**Affiliations:** 1 Department of Anesthesiology, Critical Care, and Surgery, Duke University School of Medicine, Durham, NC, USA; 1 Department of Anesthesiology, Critical Care, and Surgery, Duke University School of Medicine, Durham, NC, USA; 2 Department of Emergency and Disaster Medicine, Juntendo University Graduate School of Medicine, Tokyo, Japan; 2 Department of Emergency and Disaster Medicine, Juntendo University Graduate School of Medicine, Tokyo, Japan; 3 Faculty of Medical Science, Juntendo University, Chiba, Japan; 3 Faculty of Medical Science, Juntendo University, Chiba, Japan

**Keywords:** sepsis, ferroptosis, damage-associated molecular patterns, NETosis, thromboinflammation

## Abstract

**Background:**

Sepsis is characterized by dysregulated inflammation and thromboinflammation in which regulated cell death and damage-associated molecular patterns (DAMPs) contribute to organ injury. Ferroptosis has recently emerged as a potential mechanism of inflammatory cell injury in sepsis.

**Objective:**

To investigate ferroptosis-associated leukocyte injury and inflammatory lytic cell death in an *in vitro* endotoxin model.

**Methods:**

Rat peritoneal leukocytes were stimulated with lipopolysaccharide (LPS; 0.4 mg/mL). Intracellular, mitochondrial, and lysosomal Fe^2+^ accumulation was evaluated using FerroOrange^®^, Mito-FerroGreen^®^, and Lyso-Ferro Red^®^ fluorescence probes. Cellular morphology was assessed using May-Grünwald-Giemsa staining, DAPI staining, and immunofluorescence for histone H3 and citrullinated histone H3.

**Results:**

LPS stimulation induced marked leukocyte injury characterized by membrane rupture, cytoplasmic collapse, and extracellular dispersion of cellular contents. Fluorescence imaging demonstrated increased intracellular, mitochondrial, and lysosomal Fe^2+^ accumulation, suggesting ferroptosis-associated iron dysregulation. DAPI staining showed chromatin injury and extracellular DNA release. Histone H3-positive and citrullinated histone H3-positive cells coexisted within the same inflammatory population, indicating overlap between inflammatory lytic cell death and NETosis-like responses.

**Conclusions:**

Ferroptosis-associated iron accumulation, DAMP release, and overlapping inflammatory cell death phenotypes may collectively contribute to thromboinflammation and organ injury in sepsis.

## Introduction

Sepsis is a life-threatening syndrome caused by a dysregulated host response to infection and remains one of the leading causes of mortality in critically ill patients. Beyond excessive inflammation alone, contemporary understanding recognizes sepsis as a complex network involving immune dysregulation, endothelial injury, metabolic stress, coagulation abnormalities, and thromboinflammation^1, 2)^. In this setting, leukocyte injury and regulated cell death are increasingly understood to play active roles in disease progression rather than representing passive terminal events^3, 4)^.

Among the various regulated cell death pathways implicated in sepsis, apoptosis has traditionally been regarded as the dominant mechanism associated with immune exhaustion and late-phase immunosuppression^5, 6)^. However, accumulating evidence indicates that inflammatory lytic forms of cell death, including pyroptosis, necroptosis, ferroptosis, and NETosis, also contribute substantially to early inflammatory amplification and organ dysfunction^3, 7)^. Unlike apoptosis, these lytic pathways are characterized by membrane disruption and extracellular release of intracellular molecules that can amplify inflammation^8)^.

Ferroptosis is a regulated cell death pathway characterized by iron-dependent lipid peroxidation and oxidative membrane injury^9, 10)^. Distinct from apoptosis and classical necrosis, ferroptosis is associated with dysregulated intracellular iron metabolism, mitochondrial dysfunction, and reactive oxygen species generation^11, 12)^. Recent experimental studies have suggested that ferroptosis contributes to organ injury in sepsis, including acute lung injury, hepatic dysfunction, renal injury, and endothelial damage^13)^. However, the role of ferroptosis in inflammatory leukocyte death remains incompletely understood.

An important consequence of inflammatory lytic cell death is the release of damage-associated molecular patterns (DAMPs), including extracellular histones, mitochondrial DNA, HMGB1, nucleosomes, and other intracellular molecules^5, 14)^. These endogenous danger signals activate pattern-recognition receptors such as Toll-like receptors and receptor for advanced glycation end products (RAGE), thereby propagating leukocyte activation, endothelial dysfunction, platelet activation, and immunothrombosis^15)^. In sepsis, DAMP-mediated thromboinflammation is increasingly recognized as a central mechanism linking cellular injury to disseminated intravascular coagulation and multiorgan failure^16)^.

Neutrophil extracellular traps (NETs) represent another important component of inflammatory cell injury in sepsis. NET formation involves chromatin decondensation and extracellular release of DNA and histones, which may trap pathogens but can also promote endothelial injury, platelet aggregation, and microvascular thrombosis^17, 18)^. Importantly, recent evidence suggests that NETosis does not necessarily occur as an isolated and independent cell death program. Instead, inflammatory leukocyte death in sepsis may involve overlapping and heterogeneous phenotypes in which ferroptosis- associated injury, lytic degeneration, pyroptosis- like membrane rupture, and NET-associated chromatin release coexist within the same inflammatory environment^19, 20)^.

Although ferroptosis has attracted increasing attention in sepsis research, direct morphological evidence linking ferroptosis-associated iron accumulation to inflammatory leukocyte injury and DAMP release remains limited. Furthermore, the relationship between ferroptosis-associated injury and NETosis-like chromatin release remains insufficiently characterized.

Therefore, the present study aimed to establish an *in vitro* endotoxin-induced leukocyte injury model and to investigate ferroptosis-associated intracellular ferrous iron (Fe^2+^) accumulation together with morphological evidence of inflammatory lytic cell death. Using fluorescent Fe^2+^ probes, May- Grünwald-Giemsa (MG) staining, DAPI staining, and immunofluorescence analysis of histone H3 and citrullinated histone H3, we sought to characterize the heterogeneity and overlap of inflammatory cell death phenotypes induced by lipopolysaccharide (LPS) stimulation.

## Materials and Methods

### Materials

Male Wistar rats (4 weeks old, 85-100 g) obtained from Tokyo Experimental Animals (Tokyo, Japan) were used in this study. Under anesthesia, animals were euthanized by cervical dislocation prior to leukocyte isolation. All experimental procedures were performed in accordance with the guidelines for animal experimentation of the Japanese Association for Laboratory Animal Science and approved by the Animal Review Board of Juntendo University (#1823).

### Isolation of peritoneal leukocytes

Peritoneal leukocytes were collected by intraperitoneal lavage using Hanks’ Balanced Salt Solution without calcium and magnesium (HBSS; STEMCELL Technologies, Tokyo, Japan). HBSS was injected into the peritoneal cavity, and the abdomen was gently massaged approximately 20 times before the lavage fluid was collected into centrifuge tubes. Glycogen induction was not performed. Collected cells were centrifuged at 230 × g for 5 min, washed twice with HBSS, and resuspended in serum-free Opti-MEM medium (Gibco, Waltham, MA, USA). Cell counts were determined, and suspensions were adjusted according to each experimental condition.

### Cell seeding and culture conditions

For ferroptosis-associated Fe^2+^ imaging, leukocytes were adjusted to 1 × 10^5^ cells/well in 200 μL Opti-MEM and seeded into 35-mm glass-bottom dishes (MatTek Corporation, Ashland, MA, USA). For immunofluorescence staining and MG staining, leukocytes were adjusted to 3 × 10^5^ cells/well in 200 μL and seeded onto glass-bottom dishes or coverslips, respectively. Cells were incubated at 37°C in a humidified atmosphere containing 5% CO_2_ to allow adhesion. Non-adherent cells were removed by washing with Opti-MEM.

### LPS-induced septic cell model

To establish an in vitro septic cell model, leukocytes were stimulated with LPS (Escherichia coli O127:B8, Sigma-Aldrich, St. Louis, MO). For ferroptosis experiments, cells were treated with LPS at a final concentration of 0.4 mg/mL for 2.5 h following a 50-min adhesion period. Untreated leukocytes served as normal controls.

### Fluorescent detection of intracellular, mitochondrial, and lysosomal Fe^2+^

Intracellular Fe^2+^ was visualized using FerroOrange^®^ (Dojindo Laboratories, Kumamoto, Japan), mitochondrial Fe^2+^ using Mito-FerroGreen^®^ (Dojindo Laboratories), and lysosomal Fe^2+^ using Lyso-Ferro Red^®^ (Dojindo Laboratories). After LPS treatment, cells were washed three times before staining. FerroOrange^®^ working solution (10 μmol/L) was added and incubated for 30 min, followed by direct imaging without washing. Mito-FerroGreen^®^ working solution (25 μmol/L) was incubated for 30 min, followed by three washes before imaging. Lyso-Ferro Red^®^ working solution (5 μmol/L) was incubated for 30 min, followed by three washes before imaging.

### May-Grünwald-Giemsa staining

For morphological evaluation, methanol-fixed samples were briefly rinsed in distilled water and stained with May-Grünwald solution (Muto Pure Chemicals, Tokyo, Japan) for 1.5 min. Samples were then overlaid with an equal volume of pH 6.4 phosphate buffer for 1.5 min, rinsed again, and stained with 10% Giemsa solution (Merck/Sigma-Aldrich) for 10 min. After washing, dehydration, and mounting with Mount-Quick medium, specimens were observed under bright-field microscopy.

### Immunofluorescence staining of histone H3 and citrullinated histone H3

Fixed leukocytes were permeabilized with 0.3% Triton X-100 in PBS for 10 min at room temperature and blocked with 5% bovine serum albumin (BSA) in PBS for 30 min. Primary antibodies against histone H3 (Cell Signaling Technology, Danvers, MA, USA) and citrullinated histone H3 (Abcam, Cambridge, UK) were diluted 1:100 in 1% BSA/0.3% Triton X-100/PBS and incubated overnight at 4°C in the dark. After washing with PBS, Alexa Fluor 488-conjugated goat anti-rabbit IgG and Alexa Fluor 594-conjugated donkey anti-mouse IgG secondary antibodies (Abcam) were applied at a dilution of 1:500 for 1 h at room temperature. Nuclear DNA was counterstained with DAPI solution (Dojindo Laboratories; 1:4000 dilution) for 10 min. Samples were mounted and observed using GFP- HQ, Texas Red, and DAPI fluorescence filter sets.

### Microscopy and image acquisition

Fluorescence microscopy was performed immediately after staining procedures. FerroOrange^®^ and Lyso-Ferro Red^®^ fluorescence signals were detected using a Texas Red filter set (Ex/Em: 565/613 nm), whereas Mito-FerroGreen^®^ fluorescence was observed using a FITC-A filter set (Ex/Em: 488/530 nm). Bright-field and fluorescence images were acquired under identical imaging conditions for comparison between the control (normal) and treated groups.

### Measurement of DAMPs

Culture medium samples (n = 4) were collected at 1 and 3 h after LPS treatment and stored at −20 °C until analysis. Extracellular levels of histone H3 and high-mobility group box 1 (HMGB1), representative DAMPs, were quantified using commercially available sandwich enzyme-linked immunosorbent assay (ELISA) kits (Shinotest, Tokyo, Japan) according to the manufacturer’s instructions. Statistical analyses were performed using the Mann-Whitney U test or analysis of variance (ANOVA), as appropriate. *P*-value < 0.05 was considered statistically significant.

## Results

LPS-induced lytic cell death and ferroptosis-associated alterations in rat leukocytes

To investigate inflammatory cell death induced by lipopolysaccharide (LPS), rat peritoneal leukocytes were analyzed using MG staining, Fe^2+^-sensitive fluorescent probes, DAPI staining, and immunofluorescence for histone H3 and citrullinated histone H3.

### May-Grünwald-Giemsa staining

**Figure 1:**
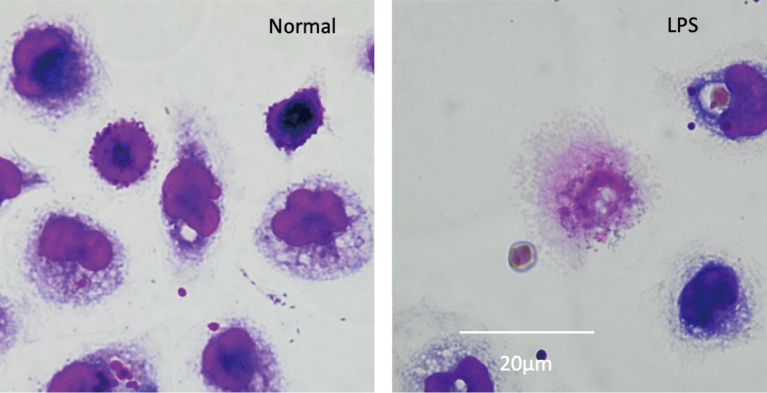
Lipopolysaccharide-induced leukocyte lytic cell death Rat leukocytes were incubated in the presence or absence of lipopolysaccharide (LPS) and subsequently stained with May-Giemsa. Under control conditions (left), leukocytes maintain a round cellular contour with preserved membrane integrity and characteristic lobulated nuclei. Following LPS exposure (right), leukocytes exhibit marked structural disruption, including membrane rupture, loss of cytoplasmic integrity, nuclear disorganization, and extracellular dispersion of cellular contents, consistent with inflammatory lytic cell death.

Under control conditions, most leukocytes maintained intact cellular morphology with preserved plasma membranes and well-defined nuclear structures. In contrast, LPS-treated leukocytes exhibited marked structural disruption consistent with inflammatory lytic cell death. MG staining demonstrated membrane rupture, cytoplasmic collapse, nuclear disorganization, and extracellular dispersion of cellular contents. In particular, disruption of plasma membrane integrity strongly suggested leakage of intracellular damage-associated molecular patterns (DAMPs) into the extracellular environment. LPS-treated leukocytes also exhibited increased cytoplasmic vacuolation and the appearance of fragmented or ghost-like cellular structures, indicating severe degenerative changes (Figure 1).

### Ferroptosis staining

**Figure 2:**
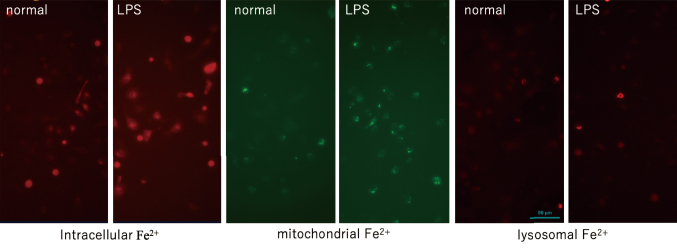
Fluorescent detection of intracellular, mitochondrial, and lysosomal Fe^2+^ in leukocytes following LPS stimulation Representative fluorescence images of leukocytes isolated from Wistar rats under normal conditions (Normal) or after stimulation with lipopolysaccharide (LPS; 0.4 mg/mL for 2.5 h). Intracellular Fe^2+^ was detected using FerroOrange, mitochondrial Fe^2+^ using Mito-Ferro Green, and lysosomal Fe^2+^ using Lyso-Ferro Red. LPS-treated cells showed increased fluorescence intensity, particularly in mitochondrial and lysosomal compartments, together with increased numbers of shrunken and damaged cells, suggesting enhanced iron accumulation associated with ferroptotic cell death.

Fluorescent Fe^2+^ staining further demonstrated intracellular iron dysregulation following LPS exposure. FerroOrange staining revealed enhanced intracellular Fe^2+^ accumulation in LPS-treated cells compared with controls. Mito-FerroGreen staining showed increased punctate fluorescence within mitochondria, whereas Lyso-Ferro Red staining demonstrated enhanced lysosomal Fe^2+^ accumulation. These findings suggest that LPS stimulation induces ferroptosis-associated iron accumulation in multiple intracellular compartments. Cells exhibiting severe structural collapse frequently showed reduced fluorescence intensity, possibly reflecting leakage or loss of intracellular contents after membrane disruption (Figure 2).

### DAPI staining

DAPI staining demonstrated diffuse and irregular nuclear morphology in LPS-treated leukocytes, consistent with chromatin decondensation and nuclear injury. Extracellular DAPI-positive material was occasionally observed surrounding damaged cells, suggesting extracellular release of DNA during lytic cell death.

### H3 and citrullinated H3 staining

Immunofluorescence analysis demonstrated coexistence of histone H3-positive cells and citrullinated histone H3-positive cells within the LPS-treated population. Histone H3 staining was broadly observed in both control and stimulated cells, whereas citrullinated histone H3 staining was more prominent after LPS stimulation. Some damaged leukocytes exhibited extracellular chromatin structures positive for citrullinated histone H3, consistent with NETosis-like chromatin release. However, not all damaged cells were positive for citrullinated histone H3, and some cells displayed morphological features of lytic degeneration without clear NET-associated staining.

**Figure 3:**
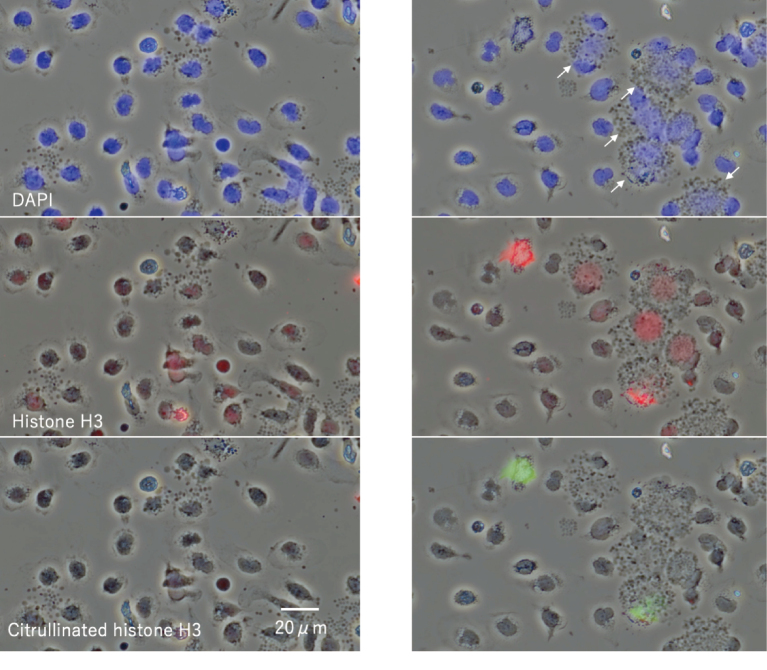
Lipopolysaccharide-induced lytic cell death in rat leukocytes Rat leukocytes were incubated in the presence or absence of lipopolysaccharide (LPS). Following alcohol fixation, nuclei were stained with DAPI (blue, upper panels). Under control conditions (left column), leukocytes maintain intact nuclear morphology and cellular structure. In contrast, LPS-treated cells (right column) demonstrate morphological features consistent with lytic cell death, including loss of cellular integrity and nuclear disorganization. A subset of LPS-exposed cells shows positive immunostaining for histone H3 and citrullinated histone H3, indicating chromatin modification associated with inflammatory activation. Citrullinated histone H3 is widely used as a marker of NET formation; however, histone citrullination is not absolutely specific to NETosis and may occur under other inflammatory conditions.

These findings suggest that LPS-induced inflammatory leukocyte death is heterogeneous and cannot be classified into distinct, completely independent categories. Instead, multiple forms of cell death, including ferroptosis-associated injury, lytic cell death, and NETosis-like chromatin release, likely coexist and overlap within the same inflammatory environment (Figure 3).

### Measurement of DAMPs

**Figure 4:**
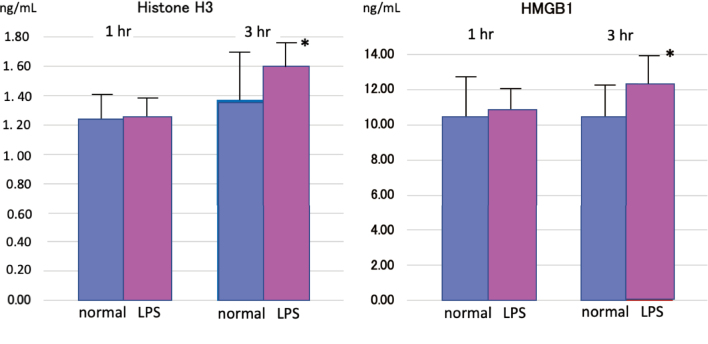
Extracellular release of DAMPs following LPS stimulation Extracellular concentrations of histone H3 and high-mobility group box 1 (HMGB1) in culture supernatants collected 1 and 3 h after lipopolysaccharide (LPS) stimulation were measured using ELISA. Both histone H3 and HMGB1 levels increased following LPS exposure and became significantly elevated at 3 h compared with untreated controls. These findings suggest that LPS-induced inflammatory leukocyte injury is associated with extracellular release of damage-associated molecular patterns (DAMPs) accompanying membrane disruption and lytic cell death. Data are presented as mean ± SD. ^*^: *P* < 0.05 versus control.

Extracellular histone H3 and HMGB1 levels increased following LPS stimulation. Histone H3 concentrations increased over time and were significantly elevated at 3 h after LPS treatment compared with untreated controls (P < 0.05). Similarly, HMGB1 levels increased after LPS exposure and reached a significant elevation at 3 h (P < 0.05). These findings indicate that LPS-induced inflammatory leukocyte injury is associated with extracellular release of DAMPs, accompanied by membrane disruption and inflammatory lytic cell death (Figure 4).

## Discussion

The present study demonstrated that LPS stimulation induces marked inflammatory leukocyte injury accompanied by intracellular Fe^2+^ accumulation, membrane disruption, extracellular chromatin release, and coexistence of histone H3-positive and citrullinated histone H3-positive cells. These findings support the concept that ferroptosis-associated injury, inflammatory lytic cell death, and NETosis-like responses coexist within the septic inflammatory environment rather than functioning as completely isolated processes.

Recent advances in sepsis biology have shifted the conceptual framework of regulated cell death from a collection of independent pathways toward a dynamic inflammatory network involving apoptosis, pyroptosis, necroptosis, ferroptosis, PANoptosis, and NET-associated injury^21)^. Inflammatory lytic cell death is increasingly recognized as a major amplifier of thromboinflammation, as membrane rupture releases extracellular DAMPs, including histones, mitochondrial DNA, HMGB1, and nucleosomes^22)^. These molecules activate pattern-recognition receptors, including Toll-like receptors and RAGE, thereby promoting leukocyte activation, endothelial dysfunction, platelet aggregation, and microvascular thrombosis^15, 22)^.

In the present study, MG staining revealed membrane rupture and extracellular leakage of intracellular contents following LPS stimulation. These morphological findings strongly suggest the release of DAMPs into the extracellular environment. Extracellular histones and DNA released from injured leukocytes are known to induce endothelial toxicity, activate coagulation pathways, and promote immunothrombosis^23)^. Thus, the observed lytic degeneration may represent an important mechanistic link between inflammatory leukocyte death and sepsis-associated thromboinflammation.

A major finding of this study was increased intracellular, mitochondrial, and lysosomal Fe^2+^ accumulation following LPS stimulation. Ferroptosis is characterized by iron-dependent lipid peroxidation and oxidative membrane injury^9)^. Although ferroptosis has been implicated in organ injury during sepsis, direct visualization of iron dysregulation in inflammatory leukocytes has remained limited. The present findings provide morphological support for ferroptosis-associated leukocyte injury in endotoxin-induced inflammation.

Mitochondrial and lysosomal iron accumulation may be particularly important in septic cell injury^24)^. Mitochondrial dysfunction promotes reactive oxygen species generation and lipid peroxidation, whereas lysosomal iron accumulation may facilitate oxidative injury through Fenton chemistry^9, 14)^. Such intracellular iron dysregulation may sensitize leukocytes to inflammatory lytic degeneration and amplify DAMP release.

Another important observation was the coexistence of histone H3-positive and citrullinated histone H3-positive leukocytes. Citrullinated histone H3 is commonly regarded as a marker of NETosis; however, not all damaged leukocytes demonstrated citrullinated histone positivity^17, 25)^. Conversely, some cells exhibited severe structural degeneration without classical NET-associated morphology. These findings suggest that inflammatory leukocyte death in sepsis is heterogeneous and overlapping.

Recent conceptual frameworks propose that regulated inflammatory cell death pathways interact extensively through shared upstream triggers including oxidative stress, mitochondrial dysfunction, inflammasome activation, and cytokine signaling^3, 21, 26)^. Therefore, ferroptosis-associated injury, pyroptosis-like membrane rupture, necrotic degeneration, and NET-associated chromatin release may occur simultaneously within the same inflammatory milieu. The present observations support this integrated model of inflammatory cell death rather than a strict separation of independent pathways.

The coexistence of ferroptosis-associated injury and NETosis-like chromatin release may also have important implications for immunothrombosis. NETs provide scaffolds for platelet adhesion and fibrin deposition, whereas extracellular histones and DAMPs induce endothelial injury and coagulation activation^23, 27)^. Accordingly, inflammatory leukocyte death may directly contribute to the formation of intravascular inflammatory thrombi and disseminated microvascular dysfunction in sepsis.

This study has several limitations. First, the experiments were performed using an *in vitro* endotoxin model and may not fully reproduce the complexity of human sepsis. Second, ferroptosis was inferred primarily from intracellular Fe^2+^ accumulation and morphological findings without direct assessment of lipid peroxidation pathways or ferroptosis inhibitors. Third, quantitative analysis of DAMP release and cytokine responses was not performed. Further studies are required to clarify the molecular signaling pathways linking ferroptosis, NETosis, and inflammatory lytic cell death in sepsis.

In conclusion, LPS-induced leukocyte injury was associated with ferroptosis-related iron accumulation, membrane rupture, extracellular chromatin release, and overlapping inflammatory cell death phenotypes. These findings support the concept that ferroptosis, NETosis-like responses, and DAMP- mediated inflammatory lytic cell death coexist within the septic inflammatory environment and may collectively contribute to thromboinflammation and organ injury in sepsis.

## Conclusion

Endotoxin-induced leukocyte injury was associated with ferroptosis-related intracellular iron accumulation, inflammatory lytic cell death, and extracellular chromatin release. LPS stimulation induced membrane disruption and leakage of intracellular contents, suggesting release of DAMPs that may further amplify thromboinflammation and organ injury in sepsis. The coexistence of histone H3-positive and citrullinated histone H3-positive cells indicates that ferroptosis-associated injury and NETosis-like responses overlap within the same inflammatory environment rather than occurring as completely independent pathways. These findings support the concept that regulated inflammatory cell death in sepsis is a heterogeneous, interconnected process involving ferroptosis, lytic degeneration, DAMP release, and NET-associated inflammation. Targeting these overlapping pathways may provide novel therapeutic approaches for the treatment of sepsis-associated thromboinflammatory organ dysfunction.

## Data availability

The data that support the findings of this study are available from the corresponding author upon reasonable request. Certain data generated using materials or proprietary reagents provided by Dojindo Laboratories are subject to restrictions and are therefore not publicly available.

## Author contributions

TI planned and performed the experiment. JHL and TI drafted the manuscript. NU, YK, and IN significantly reviewed the manuscript. All authors read and approved the final manuscript.

## Conflicts of interest statement

The authors declare that they have no conflict of interest. JHL, TI and IN are the members of the JMJ editorial board and were not involved in the peer review or decision-making process for this paper.
